# Cincinnati Academy of Medicine

**Published:** 1871-01

**Authors:** 


### EXTRACTS FROM THE PROCEEDINGS OF THE CINCINNATI ACADEMY OF MEDICINE.

STRANGULATED HERNIA.—Dr. Dawson reported a case of strangulated inguinal hernia, seen in consultation with Dr. Bramble. The patient was an adult negro. Chloroform and taxis had been repeatedly essayed; reduction was impossible from the old adhesions to the sac and the scrotum. Seventy-two hours after the first visit, the symptoms indicating the necessity, Dr. Dawson cut down upon the bowel, by careful dissection, through the superposed layers, when he suddenly found he had entered the gut. The adhesions between it and the sac were of such nature as to render them a common tissue, so that distinction was impossible. Having united the intestine with the Glover's suture, he finally succeeded in separating the bowel, and returned it into the abdomen. Death ensued in forty-eight hours. The case was one of old, irreducible hernia, and though the patient claimed to have himself returned it on several occasions, it was highly improbable that this result had ever been entirely accomplished. Dr. Dawson commented at some length upon the individuality of every case in practice.

*Dr. Gobrecht* having excluded cases of the character of the present report, remarks that he has been entirely successful in effecting reduction by means of cold. He explains its action by its known power of exciting contraction of the blood vessels and intestinal walls, thus diminishing their size. Heat and relaxants have a contrary effect. The first case of its employment in his observation was at the Pennsylvania Hospital, eighteen years ago.

It was then stated that prolonged taxis should be avoided, from the danger of exciting inflammation. The ice bag was applied, and speedy reduction effected. Since this time he has himself successfully employed it in five or six cases.

*Dr. Conner* suggests that, had there been no adhesion to the scrotum, the hernia might have been reduced *en masse*. Such adhesion as existed in this case, however, precluded the attempt. He further relates the case of a soldier who was suddenly awakened in the night by the pain of an immense tumor at the external ring. Taxis failed. There were no constitutional effects for four days, when death suddenly supervened. A decided reaction was manifest, however, a few hours before its occurrence. A *post mortem* revealed the bowel divided by the constriction for three-fourths of an inch of its circumference. The feces being removed it could readily be returned. Dr. Conner remarks further upon the great efficacy of cold, detailing a case of recent success in his own practice, in an infant with double rupture. The value of morphia hypodermically in certain cases, and the reputed efficacy of acet. zinc in 5-15 grs., with opium 1 gr., per annum, were also suggested.

*Dr. Gobrecht* hoped that his remarks had not created the impression that he did not believe in the occasional necessity for the knife. The constriction may be at the neck of the sac, and then even a reduction *en bloc* would not afford relief.

*Dr. Dawson* repeats that the general adhesions to the scrotum prevented a reduction *en masse*. Gross states that he has not used the knife since the introduction of chloroform, but in a case of this kind incision offered the only hope of relief.

*Dr. Gobrecht* reports a case of hernia of the sigmoid flexure, in which forty-eight hours of taxis excited such inflammation as to produce one and a half pints of pus in the peritoneal cavity. The protrusion exceeded any case of his observation.

*Dr. Ludlow* stated that two years ago he reported a case of strangulated oblique inguinal hernia, wherein he succeeded in dilating the constriction in forty minutes, by introducing the little finger, followed successively by others, between the gut and the seat of constriction, as recommended by Gross and Erichsen.

Upon this a discussion sprang up as to the possibility of entering the finger, and of effecting dilation of fibrous tissue in general. The discussion was maintained by Drs. Conner, Ludlow, Gobrecht and Orr.

*Dr. Carson* spoke of a case of Dr. Blackman's, somewhat similar to that reported by Dr. Dawson. It was an old hernia, with several diverticula and some incarceration.

*Dr. Dawson* says, in regard to the return of a hernia, *en bloc*, that symptoms of strangulation have persisted even after its performance. The surgeon, in such case, has cut down upon it and divided the stricture with complete relief. Dr. Wood had just such a case.—*Cincinnati Lancet & Observer.*

